# Supplementary material for: Kiwifruit Resistance to Sclerotinia sclerotiorum and Pseudomonas syringae pv. actinidiae and Defence Induction by Acibenzolar-S-methyl and Methyl Jasmonate Are Cultivar Dependent
Source: Int J Mol Sci. 2023 Nov 3;24(21):15952. doi: 10.3390/ijms242115952 (PMC10647243; doi:10.3390/ijms242115952)
Supplement: Supplementary file 1 [file ijms-24-15952-s001.zip › ijms-2664399-supplementary.pdf]

International Journal of
Molecular Sciences

MDPI

Supplementary materials

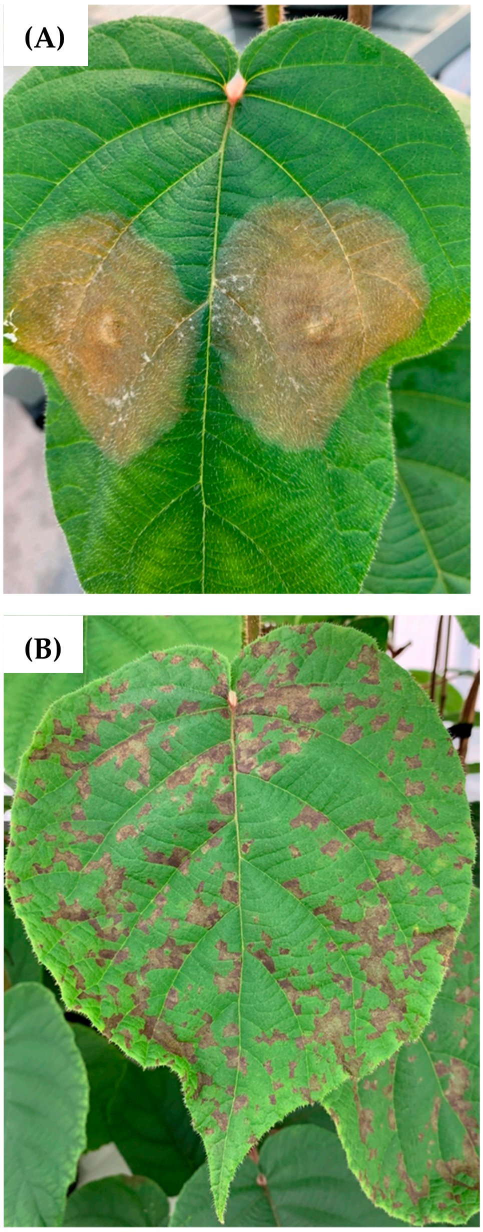

Figure S1. Symptoms of infection in *Actinidia chinensis* var. *deliciosa* 'Hayward' leaves following inoculation with A) *Sclerotinia sclerotiorum*, and B) *Pseudomonas syringae* pv. *actinidiae* (Psa biovar3).
